# Editorial: Skeletal Muscle Immunometabolism

**DOI:** 10.3389/fphys.2021.683088

**Published:** 2021-04-28

**Authors:** Capucine Trollet, Arthur J. Cheng, Lykke Sylow, Miguel L. Batista, Nicolas J. Pillon

**Affiliations:** ^1^Sorbonne Université, Inserm, Institut de Myologie, Centre de Recherche en Myologie, Paris, France; ^2^Muscle Health Research Centre, Faculty of Health, School of Kinesiology and Health Science, York University, Toronto, ON, Canada; ^3^Department of Nutrition, Exercise and Sports, Faculty of Science, University of Copenhagen, Copenhagen, Denmark; ^4^Department of Biochemistry, Boston University School of Medicine, Boston, MA, United States; ^5^Department of Physiology and Pharmacology, Karolinska Institutet, Stockholm, Sweden

**Keywords:** immunometabolism, skeletal muscle, inflammation, exercise, cachexia, metabolism, skeletal muscle atrophy, myokines

## Introduction

Skeletal muscle inflammation is triggered by multiple physiological and pathological processes. Resident immune cells such as macrophages and dendritic cells respond to the inflammatory state of skeletal muscle, and circulating immune cells can be recruited to muscle tissue (Pillon et al., 2013). Skeletal muscle adaptation depends on sequential stages of degeneration, inflammation, and regeneration (Chazaud, 2016). This remodeling process results from a finely tuned orchestration of cellular, molecular and metabolic responses involving both muscle and non-muscle cells (inflammatory cells, endothelial cells, fibro-adipogenic cells, pericytes). If unbalanced, this response leads to muscle atrophy, and/or fibrosis. This Research Topic on immunometabolism incorporates reviews and original studies to elucidate the many implications of skeletal muscle inflammation in metabolism, health, and disease.

## Skeletal Muscle-Immune Crosstalks

The inflammatory response needed for optimal muscle adaptations involves crosstalk between the immune and non-immune cells. Bonomo et al. summarize the interactions between macrophages, dendritic, and T-cells in inflammatory responses associated with idiopathic inflammatory myopathies, Duchenne muscular dystrophy, and muscle regeneration. The review by Howard et al. describes the orchestration of the inflammatory response mediated by satellite cells and immune cells during skeletal muscle recovery from injury. The activation state of immune cells attracted to skeletal muscle is paramount to adequately trigger pro- or anti-inflammatory responses, and the use of glycolysis or fatty oxidation as the primary energy source influences this activation (Van den Bossche et al., 2017). Based on this concept, Rousseau et al. reduced fatty acid oxidation selectively in T cells by deleting the Peroxisome Proliferator-Activated Receptor beta/delta (PPARβ/δ). Deletion of PPARβ/δ in T cells increased the infiltration of T cells during skeletal muscle regeneration and prevented the age-induced decline in lean mass and endurance capacity. These effects are likely due to the inability of T cells to adjust their activation state. These three articles emphasize the role of resident and infiltrating immune cells in promoting skeletal muscle regeneration and maintenance.

## Skeletal Muscle As A Secretory Organ

Skeletal muscle is able to release “myokines” and small molecules to other types of cells and organs, with autocrine, paracrine, and long-distance endocrine effects. Bay and Pedersen discuss the role of skeletal muscle as a secretory organ, particularly regarding cytokines and growth factors acting on remote tissues such as adipose tissue, pancreas, liver, gut, and brain. Rogeri et al. focus on the role of glutamine and the myokine IL-6 in skeletal muscle and monocyte/macrophage functions. Under metabolic stress, such as exercise or an excess of fatty acids, skeletal muscle can also release small molecules such as ATP (Groen et al., 2019), a potent attractant and activator of immune cells (Pillon et al., 2014). Cruz and Beall demonstrate that extracellular ATP released by myotubes does not mediate fatty acid-induced insulin resistance but acts on myocytes to improve glucose uptake and glycolysis. These three studies illustrate the multiple inflammatory and metabolic roles of soluble molecules released by skeletal muscle.

## Skeletal Muscle Atrophy

Loss of skeletal muscle mass has major health consequences, from decreased immunity to a higher risk of falls and fractures, leading to an increase in functional dependency and mortality (Marzetti et al., 2017). Muscle atrophy involves multiple factors including protein degradation by the ubiquitin-proteasome system (UPS). Tortola et al. reveal new regulators of the E3 ubiquitin ligase TRIM63 (MuRF1), which plays essential roles in UPS-mediated muscle atrophy. Using overexpression systems they propose the involvement of the transcription factor TFE3, protein kinase D (PKD2/3), and HDAC isoforms (HDAC-4 and HDAC-7). To promote skeletal muscle mass, Hagg et al. suggest a strategy to target the transmembrane prostate androgen-induced (TMEPAI) which inhibits the SMAD2/3 pathway. In mice, overexpression of TMEPAI increases skeletal muscle mass by as much as 30% and prevents muscle atrophy in a rodent model of cancer cachexia. With the same objective to prevent atrophy, Shen et al. find that the flavonoid isoquercitrin reduces inflammation, oxidative stress, UPS, and mitophagy, and overall protects against denervation-induced muscle mass loss. These three studies add to the current understanding of the molecular mechanisms underlying skeletal muscle atrophy.

## Skeletal Muscle Adaptation To Exercise

Optimized exercise protocols to promote muscle force and hypertrophy have a wide range of applications, from improving performance in athletes to preventing metabolic diseases and cachexia, or delaying aging-associated sarcopenia (Cartee et al., 2016). Peake et al. investigate the effects of cold water immersion on the genes and proteins regulating muscle hypertrophy following an acute bout of resistance exercise. Their findings show that post-exercise cold water immersion can blunt muscle hypertrophy irrespective of exercise-induced alterations in factors that control skeletal muscle myogenesis, proteolysis, and extracellular matrix remodeling. Although this study did not directly look at this, it is plausible that cold water immersion would affect inflammatory responses and consequently impair skeletal muscle response to exercise (Tipton et al., 2017). Resistance exercise increases skeletal muscle inflammation, and macrophages play a critical role in the repair of skeletal muscle tissue in response to inflammation. However, in aged skeletal muscle, this tissue repair appears dysfunctional. Jensen et al. provide a 7-day time course of muscle macrophage activity and the response of downstream molecular targets following a single bout of resistance exercise. They observe a trend toward greater macrophage content in muscle biopsies from the elderly, and their findings further reveal that classically defined pro- and anti-inflammatory macrophage subtypes do not appear to exist in healthy aged skeletal muscle.

## Skeletal Muscle in Cancer Cachexia

Cachexia is characterized by extreme weight loss, muscle wasting, systemic inflammation, and severe metabolic dysregulation (Argilés et al., 2018). Webster et al. describe pro-inflammatory cytokines and cellular processes associated with cachexia and their possible contribution to skeletal muscle atrophy. Focusing on the skeletal muscle microenvironment, VanderVeen et al. provide insights into the integrated networks of responses between immune cells, satellite cells, fibroblast cells, and endothelial cells and their regulatory role on myofiber size and plasticity. In mice, VanderVeen et al. demonstrate that the chemotherapy drug 5-fluorouracil can contribute to muscle wasting by depleting skeletal muscle immune cell populations. They demonstrate that infiltrating and resident immune cells in skeletal muscle are disrupted due to a sensitivity of skeletal muscle to the off-target effects of 5-fluorouracil.

Physical inactivity is commonly associated with cancer and contributes to muscle wasting. Yamada et al. describe that cancer-induced and inactivity-induced muscle atrophy are regulated by different mechanisms. In a preclinical mouse model of cancer cachexia, cancer exacerbated muscle wasting in denervated skeletal muscles, due to selective myosin loss, increased autophagy, and decreased protein synthesis. On the opposite, Leal et al. review the benefits of exercise training in cancer cachexia. Cellular and biochemical mechanisms by which exercise may counter cancer cachexia are discussed, as well as the challenges to the application of exercise protocols in clinical practice. These articles provide insights into the inflammatory state of skeletal muscle during cancer cachexia and the role of exercise as a countermeasure to prevent muscle mass loss.

## Skeletal Muscle in Metabolic Diseases

Obesity and type 2 diabetes are associated with a chronic state of inflammation. Under metabolic stress, activated immune cells infiltrate the adipose, liver, and skeletal muscle tissues, a mechanism contributing to the development of insulin resistance (Hotamisligil, 2017). In a transcriptomic meta-analysis, Manti et al. compare the signature of skeletal muscle in women with obesity and polycystic ovary syndrome (PCOS), a condition associated with metabolic dysfunction in women of reproductive age. They find a negative enrichment in inflammatory pathways, suggesting impaired immune function in skeletal muscles from women with PCOS. Both obesity and PCOS are associated with insulin resistance, which highlight the context-dependent ambivalent roles of the immune system on whole-body metabolism.

## Perspectives

Inflammation is a key element of skeletal muscle adaptation to pathophysiological stresses, and which involves cellular (pro- and anti-inflammatory monocyte/macrophages, dendritic cells, T cells), and molecular actors (IL-6, TNFα, TGFβ, and TWEAK) that largely depend on whole-body homeostasis. An appropriate response involves an adequate and timely expression of inflammatory molecules (Chazaud, 2016). Elevated/uncontrolled inflammation leads to deleterious skeletal muscle adaptations and contributes to sarcopenia, cachexia, and metabolic diseases. This is also the case in several muscular dystrophies where inflammation, fibrosis, and/or muscle atrophy are major complications, often due to continuous muscle fiber breakdown (Serrano and Muñoz-Cánoves, 2017). In the context of muscular dystrophies, future directions will have to include combined approaches to holistically treat the primary genetic cause but also these secondary consequences (Cordova et al., 2018). More generally, future directions to finely tune muscle inflammation should not only include local pro- or anti-inflammatory strategies but should also consider holistic approaches to improve the overall skeletal muscle homeostasis through exercise, nutrition, as well as regulation of the immune system and metabolism. Future studies are needed to further understand the skeletal muscle immunometabolic signature in each of these contexts.

## Author Contributions

CT, AC, LS, MB, and NP contributed to the writing and editing of the manuscript. NP organized the work and finalized the manuscript. This manuscript has been approved by all named authors.

## Conflict of Interest

The authors declare that the research was conducted in the absence of any commercial or financial relationships that could be construed as a potential conflict of interest.
